# Efficacy and safety of therapies for COVID-19 in pregnancy: a systematic review and meta-analysis

**DOI:** 10.1186/s12879-023-08747-2

**Published:** 2023-11-09

**Authors:** Francesco Di Gennaro, Giacomo Guido, Luisa Frallonardo, Francesco Vladimiro Segala, Rosalba De Nola, Gianluca Raffaello Damiani, Elda De Vita, Valentina Totaro, Mario Barbagallo, Emanuele Nicastri, Antonella Vimercati, Ettore Cicinelli, Giuseppina Liuzzi, Nicola Veronese, Annalisa Saracino

**Affiliations:** 1 Clinic of Infectious Diseases, Department of Precision and Regenerative Medicine and Ionian Area - (DiMePRe-J), University of “Aldo Moro”, University of Bari “Aldo Moro”, Piazza Giulio Cesare N. 11 Cap 70124, Bari, Italy; 2 Clinic of Obstetrics & Gynaecology, University of Bari “Aldo Moro”, Bari, Italy; 3 Geriatrics Section, Department of Internal Medicine, University of Palermo, Palermo, Italy; 4 National Institute for Infectious Diseases ‘Lazzaro Spallanzani’ (IRCCS), Rome, Italy

**Keywords:** COVID-19, Pregnancy, SARS-CoV-2, Monoclonal antibodies, Antivirals, Maternal morbidity

## Abstract

**Background:**

Clinical evidence suggests that pregnant women are more vulnerable to COVID-19, since they are at increased risk for disease progression and for obstetric complications, such as premature labor, miscarriage, preeclampsia, cesarean delivery, fetal growth restriction and perinatal death. Despite this evidence, pregnant women are often excluded from clinical trials, resulting in limited knowledge on COVID-19 management. The aim of this systematic review and meta-analysis is to provide better evidence on the efficacy and safety of available COVID-19 treatment in pregnant women.

**Methods:**

Four authors searched major electronic databases from inception until 1 st November-2022 for controlled trials/observational studies, investigating outcomes after the administration of anti-SARS-CoV-2 treatments in pregnant women affected by COVID-19. The analyses investigated the cumulative incidence of delivery and maternal outcomes in pregnant women, comparing those taking active medication vs standard care. Risk ratios (RRs) with 95% confidence intervals were calculated. Statistical significance was assessed using the random effects model and inverse-variance method. This systematic review and meta-analysis was conducted in accordance with the updated 2020 Preferred Reporting Items for Systematic Reviews and Meta-Analyses (PRISMA) guidelines. The protocol has been registered in Prospero (number registration: CRD42023397445).

**Results:**

From initially 937 non duplicate records, we assessed the full texts of 40 articles, finally including ten studies. In six studies, including 1627 patients, the use of casirivimab/imdevimab (CAS/IMD), remdesivir, and IFN-alpha 2b significantly decreased the need of cesarean section ((RR = 0.665; 95%CI: 0.491–0.899; *p* = 0.008; I 2 = 19.5%;) (Table 1, (Fig. 1). Treatments did not decrease the risk of preterm delivery, admission to neonatal ICU, or stillbirth/perinatal loss (*p*-values > 0.50 for all these outcomes) and did not prevent the progression of disease towards severe degrees (k = 8; 2,374 pregnant women; RR = 0.778; 95%CI: 0.550–1.099; *p* = 0.15; I 2 = 0%). Moreover, the use of medications during pregnancy did not modify the incidence of maternal death in two studies (Table 2).

**Conclusions:**

To our analysis, CAS/IMD, remdesivir, and IFN alpha 2b reduced the number of cesarean sections but demonstrated no effect on disease progression and other obstetric and COVID-19 related outcomes. The inability to evaluate the influence of viral load on illness development in pregnant women was attributed to lack of data. In our systematic review, no major side effects were reported. Though, it is essential for the medical community to focus more on clinical trials and less on episodic case reports and case series, with standardization of fetal and maternal outcomes.

**Supplementary Information:**

The online version contains supplementary material available at 10.1186/s12879-023-08747-2.

## Introduction

The physiological changes occurring in pregnancy, e.g., immunological, respiratory, coagulative, and cardiovascular, can make pregnancy a risk factor for several courses of SARS-CoV-2 infection, both for mother and child, consequently requiring hospitalization, medical interventions, and intensive care admission [1]. This assertion is supported by clinical evidence indicating that pregnant women who have been infected with previous coronaviruses, such as Severe Acute Respiratory Syndrome (SARS) and Middle Eastern Respiratory Syndrome (MERS), were regarded as potentially more susceptible to experiencing a severe disease [2]. Pregnant women have a higher risk of miscarriage, rupture of membranes, premature prelabor, preeclampsia, cesarean delivery, fetal growth restriction and perinatal death [3].

There are other factors that could potentially clarify the underlying risk that SARS-CoV-2 poses to pregnant women and their fetuses. First, the temporary overexpression of the ACE-2 receptor in the placenta allows the virus to cause histopathological and perfusion changes, massive perivillous fibrin depositions, necrosis of syncytiotrophoblast, and diffuse chronic intervillositis, all of which could have negative consequences for the foetus and the continuation of pregnancy [4, 5].

Furthermore, the reduction of total lung capacity and the consequent hyperventilation, also due to the growing uterus, can cause one to inhale more air within the same period of time with more exposure to SARS-CoV- infection [6, 7], while also affecting the maternal immune system (an increase in maternal serum levels of toll-like receptors TLR-1 and TLR-7, and the increase of angiotensin-converting enzyme 2 [ACE-2]), which can decrease the efficacy of viral clearance [8, 9] and increase the risk of several diseases.

Furthermore, the state of hypercoagulability, which occurs primarily in the third trimester and the immediate postpartum period, is a risk factor for thrombotic events and thus contributes to the worsening of the clinical course of SARS-CoV-2 infection [10, 11].

Despite concerns about the increased vulnerability of pregnant women to COVID-19, this population remains an underrepresented group in the study of drug therapy, with pregnant people excluded from vaccine and therapy trials.

In fact, still three years after the outbreak of the COVID-19 pandemic, the choice of the appropriate treatment for pregnant patients is a relevant clinical issue that should consider the drug's safety for the patient and the fetus.

This systematic review and meta-analysis aimed to provide better evidence of COVID-19 treatment in pregnant women, in terms of efficacy and safety.

## Materials and methods

This systematic review and meta-analysis was conducted in accordance with the updated 2020 Preferred Reporting Items for Systematic Reviews and Meta-Analyses (PRISMA) guideline [12]. The protocol has been registered in Prospero (number registration: CRD42023397445).

### Search strategy

Four independent reviewers, by couples, searched PubMed, Embase, Web of Science, and Scopus from inception until 01^st^ November 2022. The full search strategy and the search terms used are the following: “(COVID-19 OR Novel Coronavirus–Infected Pneumonia OR 2019 novel coronavirus OR 2019-nCoV OR SARS-CoV-2) AND (Lopinavir OR ritonavir OR Darunavir/cobicistat OR Methylprednisolone OR Prednisone OR Hydrocortisone OR Hydroxychloroquine OR Dexamethasone OR Enoxaparin OR. Low molecular weight heparins OR Remdesivir OR Anakinra OR Baricitinib. OR Sarilumab OR Tocilizumab OR Casirivimab OR Imdevimab OR Regdanvimab. OR bamlanivimab OR etesevimab OR Sotrovimab OR Tixagevimab OR Cilgavimab. OR Nirmatrelvir OR Molnupiravir OR Favipiravir OR Colchicine OR. Chloroquine OR Nafamostat mesylate OR Camostat mesylate OR Infliximab OR. Tofacitinib OR Bebtelovimab OR Ruxolitinib OR Nitazoxanide OR. Plitidepsin OR Zotatifin OR Niclosamide OR nelfinavir OR inhibitors of HIV protease OR Hyperimmune plasma OR Interferon OR ibuprofen OR Celecoxib) AND (("Pregnancy"[Mesh] OR "Pregnant Women"[Mesh] OR pregnanc*))”. Discrepancies in the literature search process were resolved by a third investigator (N.V.). Rayyan, a free-access website, was used for title/abstract screening (https://www.rayyan.ai/).

### Inclusion and exclusion criteria

Studies were included based on the following PICO question:

- ➙Participants: pregnant women;
- ➙Intervention: pharmacological intervention for the treatment of acute SARS-CoV2 infection;
- ➙Comparison: placebo or standard of care;
- ➙Outcomes: delivery and maternal health endpoints;
- ➙Study design: randomized controlled trials, clinical controlled trials, observational studies.

Published articles were excluded if they (i) were reviews, letters, in vivo or in vitro experiments, commentaries, or conference abstracts; (ii) absence of a control group or active control group (e.g., another medication); (iii) data non meta-analyzable.

### Data extraction and risk of bias

Four authors extracted data independently which included name of first author, date of publication, country of origin, population included, type of study, follow-up (standardized in weeks), mean or median age of the women included, gestational age (weeks), number of vaccinated women, name and dosage of the intervention drugs. Disagreements between authors were resolved by one independent reviewer (N.V.).

The Newcastle–Ottawa Scale (NOS) was used to assess the study quality/risk of bias [13]. The NOS assigns a maximum of 9 points based on three quality parameters: selection, comparability, and outcome. The evaluation was made by one investigator (FVS) and checked by another (NV), independently. The risk of bias was consequently categorized as high (< 5/9 points), moderate (6–7), or low (8–9) [14].

### Outcomes

Outcomes were the evaluation of maternal, fetal, delivery and neonatal outcomes according to the International Federation of Gynecology and Obstetrics (FIGO) classification. (Safe Motherhood and Newborn Health Committee. FIGO Consensus Guidelines on Intrapartum Fetal Monitoring. Available online: https://www.jsog.or.jp/international/pdf/CTG.pdf (accessed on 25 September 2022) [15]. The outcomes of our interest were divided in delivery outcomes, i.e., preterm delivery, Cesarean section, admission to neonatal ICU (intensive care unit), stillbirth/perinatal loss, obstructed labor and maternal outcomes, i.e., COVID-19 progression to severe disease (admission to ICU, respiratory failure and need for invasive ventilation, involvement of multiple organ systems), maternal death, miscarriage/fetal loss, amniotic fluid complications, ectopic pregnancy, placental complications, eclampsia or pre-eclampsia, severe bleeding, obstetric fistula, infections.

### Statistical analysis

The analyses investigated the cumulative incidence of delivery and maternal outcomes in pregnant women comparing those taking an active medication vs standard care. We calculated the risk ratios (RRs) with their 95% confidence intervals (CIs), Statistical significance was assessed using the random effects model and inverse-variance method [12, 16].

Statistical heterogeneity of outcome measurements between different studies was assessed using the s I^2^. The classification of data as having low heterogeneity was based on I^2^ from 30 to 49%, moderate heterogeneity from 50 to 74%, and high heterogeneity from 75% and above [17]. In case of high heterogeneity and having at least 10 studies for an outcome [17], we plan to run a meta-regression analysis to explore potential sources of variability that could affect estimate rates among studies [18].

Publication bias was assessed by visually inspecting funnel plots and using the Egger bias test [19]. In case of statistically significant publication bias, the trim-and-fill analysis was planned [20]. For all analyses, a *P*-value less than 0.05 was considered statistically significant. All analyses were performed using STATA version 14.0 (StataCorp).

## Results

### Literature search

**Fig. 1:**
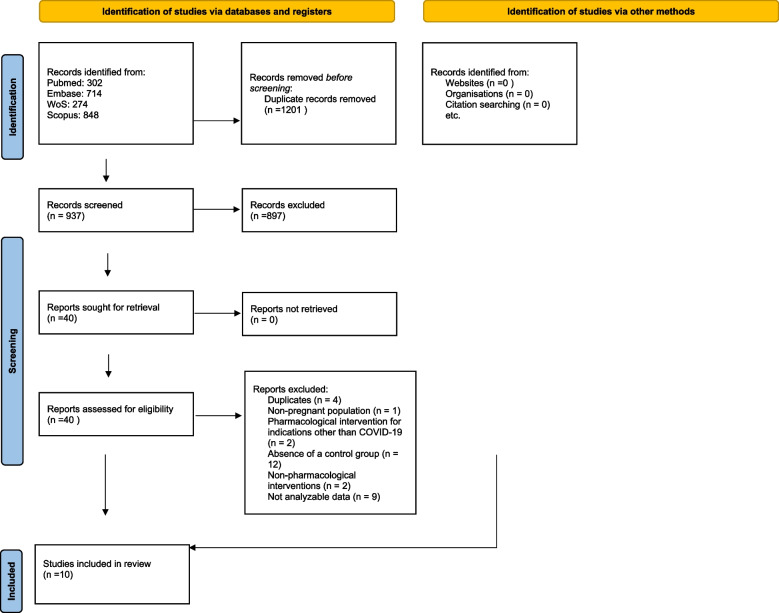
Flow-chart of the studies included

The literature search is fully reported in the PRISMA flow-chart (Fig. 1). We initially identified 937 non duplicate records. After excluding 897 works from their titles and abstracts, we assessed the full-texts of 40 articles, finally including ten studies [19–28]. The absence of a control group was the main reason of the exclusion after full-text screening.

### Descriptive findings

Table 1 shows the descriptive characteristics of the studies included. Overall, 2,463 pregnant women with a mean/median age of 30 years were followed-up for an average period of three weeks. Their mean gestational age was 25 weeks (range: 6 to 29.8 weeks). Overall, only seven studies reported the vaccination status, with about one third of the pregnant women being vaccinated against COVID-19 (36.7%). Regarding the study design, only one had a prospective design [20], whilst the other studies had a retrospective or case–control design. Among the interventions used, the drug most used was the combination of casirivimab/imdevimab (four studies), followed by remdesivir (two studies) and IFN alpha 2b (two studies) (Table 1). However, no outcome had a high heterogeneity.

**Table 1:** Descriptive characteristics for the studies included

Author, year	Population	Type of study	Follow-up (weeks)	Mean/median age (years)	Gestational age (weeks)	Number of vaccinated	Drug used as intervention	Dosage of the intervention drug (number-unit)	Quality of the studies (0–9)
Nasrallah, 2021 [20]	hospitalized women with moderate COVID-19	Prospective cohort	8	32 (range 16–44)	29.2	NR	Remdesivir	200 mg day 1 + 100 mg days 2–5	5
Kravchenko, 2021 [27]	hospitalized women with moderate COVID-19	Case–control study	NA	31.18 (range NA)	27.7	NA	IFN alpha 2b	3,000,000 UI rectal suppositories bid + 4000 UI gel five times/day on the nasal mucosa	6
Magawa, 2022 [29]	hospitalized women with moderate COVID-19	Retrospective cohort	0.7	30.25 (range 20–37)	28	0/8	Casirivimab/ imdevimab	600 mg/600 mg	7
Eid, 2022 [30]	non-hospitalized women with mild COVID-19	Retrospective cohort	NA	29.2 ± 8.2	26	43/115	Casirivimab/ imdevimab	NR	5
Valsecchi, 2022 [31]	hospitalized women with severe COVID-19	Retrospective cohort	4	30.3 ± 4.43	29.77	0/20	Nitric Oxide	200 ppm × 2	6
Tumash, 2022 [28]	hospitalized women with moderate COVID-19	Retrospective cohort	NA	33 (range NA)	32	NA	Remdesivir	200 mg day 1 + 100 mg days 2–5	5
Sinchikhin, 2022 [25]	non-hospitalized pregnant women exposed to COVID-19	Prospective cohort	3	25 (range NA)	NR	7/37	IFN alpha 2b	5000 ui	6
Levey, 2022 [32]	non-hospitalized pregnant women positive or exposed to COVID-19	Retrospective cohort	0	29.2 (range NA)	NR	0/36	Casirivimab/ imdevimab	600 mg/600 mg	5
Williams, 2022 [33]	non-hospitalized women with mild COVID-19	Retrospective cohort	NA	NA	NA	0/88	Casirivimab/ imdevimab	600 mg/600 mg	6
McCreary, 2022 [34]	non-hospitalized women with mild COVID-19	Retrospective cohort	4	30 (range 26- 33)	6	265/552	Bamlanivimab and etesevimab. casirivimab and imdevimab. or sotrovimab treatment compared with no mAb treatment	NA	7

NA: not applicable, NR: not reported

### Efficacy and safety of medications in pregnant women on delivery outcomes

**Fig. 2:**
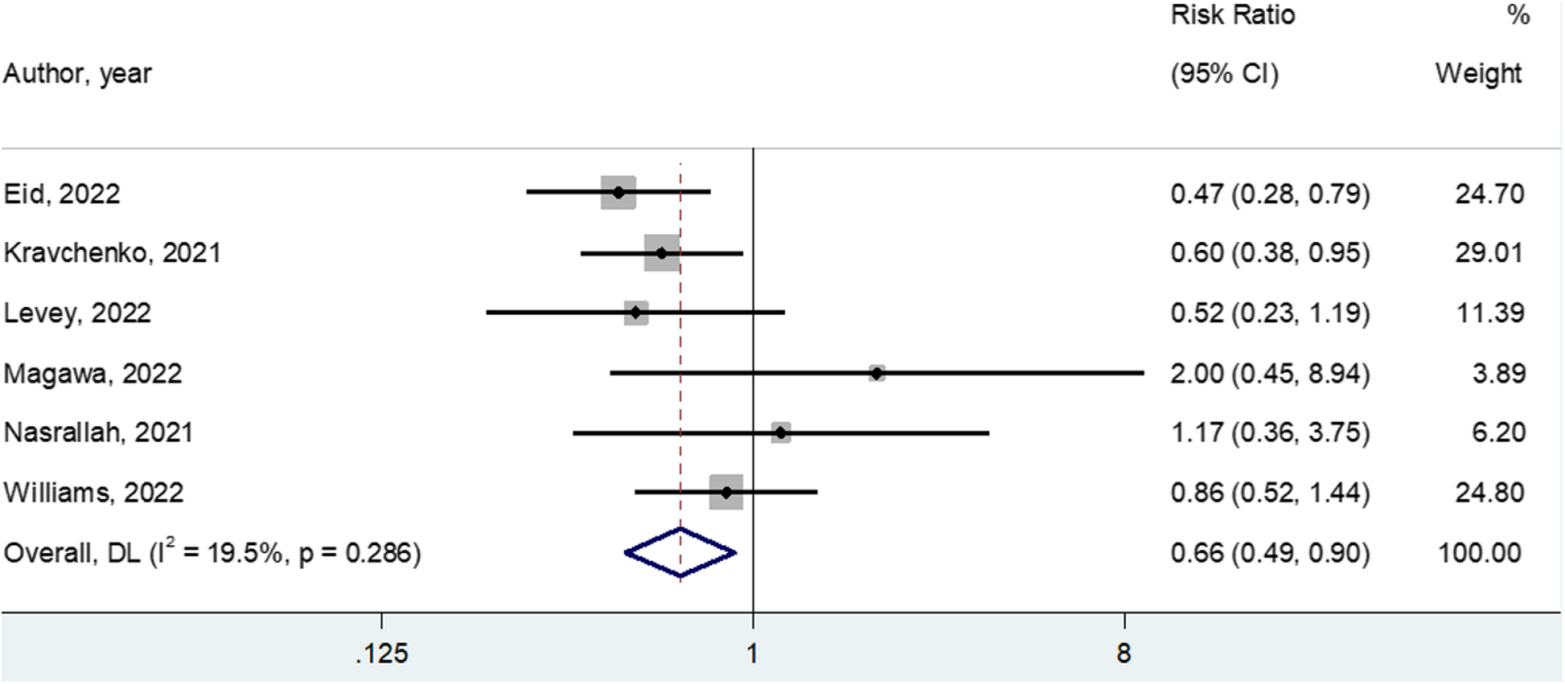
Meta-analysis of medications versus standard care in preventing Cesarean section

Table 2 shows the effect of any medication on delivery outcomes. In particular, in six studies including 1627 pregnant women, the use of casirivimab/imdevimab (four studies), remdesivir (one study) and IFN alpha 2b (one study) significantly decreased the need of Cesarean section (RR = 0.665; 95%CI: 0.491–0.899; *p *= 0.008; I^2^ = 19.5%; Fig. 2 for the forest plot). When including only the studies using casirivimab/imdevimab, the re-calculated effect size was similar. This outcome did not suffer on any publication bias (Table 2). On the contrary, the use of medications did not decrease the risk of preterm delivery, admission to neonatal ICU, or stillbirth/perinatal loss (*p*-values > 0.50 for all these outcomes). Finally, no study reported data regarding obstructed labor (Table 2).

**Table 2:** Meta-analysis of the delivery outcomes for the studies included

Outcome	Number of studies	Sample size	Risk ratio (95% CI)	*p*-value	I2	Egger’s test (SE), *p*-value
**Cesarean section**	6	1627	0.665	0.008	19.5	1.62 (1.10) *P* = 0.22
			(0.491–0.899)			
**Preterm delivery**	7	2501	0.874	0.50	43.5	-1.09 (1.17) *P* = 0.40
			(0.591- 1.294)			
**Admission to neonatal ICU**	4	2284	1.099	0.54	4.2	-1.47 (1.00) *P* = 0.28
			(0.810–1.490)			
**Stillbirth/perinatal loss**	4	1449	0.932	0.93	15.4	2.99 (5.19) *P* = 0.62
			(0.200–4.347)			
**Obstructed labor**	Not reported

### Efficacy and safety of medications in pregnant women on maternal outcomes

Table 3 shows the efficacy and safety of medications used in pregnancy taking maternal outcomes as endpoints. Overall, the use of medications during pregnancy did not modify progression towards severe COVID-19 (k = 8; 2,374 pregnant women; RR = 0.778; 95%CI: 0.550–1.099; *p* = 0.15; I^2^ = 0%). Moreover, the use of medications during pregnancy did not modify the incidence of maternal death in two studies. Finally, one study reported that no difference in placental complications, eclampsia or pre-eclampsia, or severe bleeding was observed (Table 3). As summarized in Table 3, no data were reported regarding other maternal outcomes of interest, i.e., miscarriage/fetal loss, ectopic pregnancy, obstetric fistula or bacterial infections.

**Table 3:** Maternal outcomes for the studies included

Outcome	Number of studies	Sample size	Risk ratio (95% CI)	*p*-value	I2	Egger’s test (SD), *p*-value
**COVID-19 progression**	8	2374	0.778	0.15	0	-0.98 (0.52) *P* = 0.11
			(0.550–1.099)			
**Maternal death**	2	1017	0.450	0.49	0	Not possible
			(0.048–4.264)			
**Miscarriage/fetal loss**	Not reported					
**Amniotic fluid complications**	1	283	0.381	0.13	Not possible	Not possible
			(0.109–1.337)			
**Ectopic pregnancy**	Not reported					
**Placental complications**	1	89	0.208	0.30	Not possible	Not possible
			(0.011–3.918)			
**Eclampsia or pre-eclampsia**	1	296	0.928	0.84	Not possible	Not possible
			(0.446 -1.929)			
**Severe bleeding**	1	280	0.310	0.13	Not possible	Not possible
			(0.068–1.407)			
**Obstetric fistula**	Not reported					
**Infections**	Not reported					

### Risk of bias

Supplementary Table 1 and Table 1 reported the evaluation of the risk of bias. Overall, the median value of the NOS was 5, indicating a poor quality of the studies included. The risk of bias, in factwas high in four studies and moderate in the other six. The most common sources of bias were the lack of comparability between treated and controls and the short follow-up.

## Discussion

In this systematic review with meta-analysis including about 2,500 pregnant women, we examined the efficacy and safety of treatments for COVID-19 infection in pregnancy.

Pregnant women constitute a unique population not only because of their increased disease risk of COVID-19 disease [9], but also in regard to the exclusion from any pre-licensure trials. Moreover, pregnant women resulted in being the one with fewer treatment options against SARS-CoV-2 during the first periods of COVID-19 pandemic, where treatment and vaccines were first approved for general population and only later for pregnant women.

In fact, over time, with the increase of efficacy of COVID-19 treatment and vaccines, it has seen a growing interest in the use and efficacy also in pregnant women as well.

Several studies have assessed the immunogenicity, safety, and efficacy of COVID-19 vaccines in pregnant women, [8] resulting in a lower risk of premature delivery and the occurrence of a small-for-gestational-age foetus in pregnant women who receive vaccinations compared to unvaccinated [21]. Additionally the safety results revealed that pregnant and breastfeeding individuals experienced mild vaccine-related local and systemic responses subsequent to the administration [22].

Additional studies with larger samples and longer follow-ups are needed to clarify the effect of multiple doses (booster included) on obstetrical and perinatal outcomes and the difference between trimesters, taking into account the time between vaccination and delivery.

The lack of treatment during pregnancy has obliged the U.S. Food and Drug Administration (FDA) to permit the emergency authorization of the administration of monoclonal antibodies (casirivimab/imdevimab or sotrovimab) against COVID-19, assessing pregnancy at high risk for severe disease as an inclusion criterion for the prescription of monoclonal antibodies [23, 24].

In our analysis, casirivimab and imdevimab (CAS/IMD) were the most commonly administered treatment in pregnant women with COVID-19 infection, resulting in a safe and effective option. From our data, CAS/IMD, together with Remdesivir and IFN alpha 2b [25], significantly reduced the need for a cesarean section, suggesting that COVID-19 medications in pregnancy might prevent or reduce vascular damage in the placenta. These data are very relevant if we evaluate that SARS-CoV-2 is responsible for a state of systemic inflammation and hypercoagulability [10], similar characteristics of pre-eclampsia that can cause abruption and might end up in a caesarean section [26]. Namely pregnant women with COVID-19 show vascular changes in placenta compared to pregnant women who did not develop infection [27].

It is assumed that the role of these treatments is to decrease viral load of SARS-CoV-2, preventing complications and worse outcomes [27, 28] this assumption is still under discussion as *Magawa *et al. [29] could not demonstrate a suppression in viral activity immediately after the administration of CAS/IMD, while *Levey *et al*. *[32] *showed a* promising reduction of viral load in patients with hypoxemia.

Nevertheless, as demonstrated by M. Gao et Al. Casirivimab-Imdevimab accelerates symptom resolution and showed considerable advantages in terms of preventing hospitalization and mortality in COVID 19 disease [35, 36]. The role of viral load reduction in viral respiratory infections during pregnancy in reducing the risk of disease progression is still under discussion as the results are discordant, considering that in our analysis the vaccination history of both groups was discontinuously known [34, 37].

Di Girolamo et al. provides in a systematic review an extensive analysis of the guidelines on management and approved therapeutic options in COVID-19 in pregnancy [38], illustrative of useful approaches regarding the timeliness of delivery and hospitalization in cases of severe SARS-CoV2 infection. Nevertheless, the finding related to the use of corticosteroids and *LMWH* provides a validation of existing knowledge [39].

Other meta-analysis have been conducted, on the evaluating the maternal fetal outcome and the effects on the placenta in SARS-CoV2 infection [40] however, the lack of standardization of these therapies highlights the need to identify opportunities for improving COVID-19 vaccination, implementation of treatment of pregnant women, and inclusion of pregnant women in clinical trials.

Further research and evidence are required to stratify pregnant populations who have received treatments that specifically target the risk reduction of COVID-19 disease.The findings of our study must be interpreted within its limitations. Beside all these observations, in our meta-analysis, there was not a significant decrease in the risk of preterm delivery, admission to the neonatal ICU, or stillbirth or perinatal loss. Both maternal and fetal outcomes need an in-depth debate since they are not standardized in the studies on COVID-19 in pregnancy, resulting in a difficult analysis of the implications of COVID-19 treatments. Furthermore, our metanalysis is meant to overcome a limitation encountered in many studies where anti-COVID-19 medications were administered to pregnant women with no comparison with a control group; this inclusion criteria has resulted in a small and heterogeneous sample size with a lack of compatibility in terms of follow-up and between the treated and control groups.

## Conclusions

In the last two years, the interest in COVID-19 in pregnant women has been progressively increasing, as has the number of publications on this topic. Nevertheless, the scientific world needs a different quality of study about COVID-19 in pregnancy, less keen on case reports and case series describing confined episodes and more keen on trials describing the real impact of SARS-CoV-2 medications on standardized maternal and fetal outcomes. COVID-19 in pregnancy is an opportunity to ask ourselves if treatments impacting viral load reduction prevent the progression towards severe disease in respiratory infections in pregnancy. According to our analysis, monoclonal antibodies and other treatments such as remdesivir and IFN alpha 2b have been demonstrated to be safe, but it is still premature to actually consider them useful to prevent worse outcomes, for both pregnant women and fetuses.

### Supplementary Information

**Additional file 1: Supplementary Table 1. **Newcastle Ottawa Scale for evaluating the risk of bias in the studies included.

## Data Availability

The study specific summary data included in the meta-analysis can be obtained from the corresponding authors at luisana.frallonardo@gmail.com.
