# Supplementary material for: Efficacy and safety of therapies for COVID-19 in pregnancy: a systematic review and meta-analysis
Source: BMC Infect Dis. 2023 Nov 9;23:776. doi: 10.1186/s12879-023-08747-2 (PMC10634005; doi:10.1186/s12879-023-08747-2)
Supplement: Supplementary file 1 — Additional file 1: Supplementary Table 1. Newcastle Ottawa Scale for evaluating the risk of bias in the studies included. [file 12879_2023_8747_MOESM1_ESM.docx]

**Supplementary Table 1. Newcastle Ottawa Scale for evaluating the risk of bias in the studies included.**

| Study ID | Representativeness of exposed cohort | Selection of the non-exposed cohort | Ascertainment of exposure | Demonstration that outcome of interest was not present at start of study | Comparability of cohorts on the basis of the design or analysis controlled for confounders | Assessment of outcome | Was follow-up long enough for outcomes to occur | | Adequacy of follow up cohorts | | TOTAL SCORE |
| --- | --- | --- | --- | --- | --- | --- | --- | --- | --- | --- | --- |
| Nasrallah et al. | 1 | 1 | 1 | 1 | 0 | 1 | 0 | | 0 | | **5** |
|  | Selection: 4 | | | | Comparability: 0 | Outcome: 1 | | | | |  |
| Magawa et al. | 1 | 1 | 1 | 1 | 0 | 1 | 1 | | 1 | | **7** |
|  | Selection: 4 | | | | Comparability: 0 | Outcome: 3 | | | | |  |
| Levey et al. | 1 | 1 | 1 | 1 | 0 | 1 | 0 | | 0 | | **5** |
|  | Selection: 4 | | | | Comparability: 0 | Outcome: 1 | | | | |  |
| Kravchenko et al. | 0 | 1 | 1 | 1 | 0 | 1 | 1 | | 1 | | **6** |
|  | Selection: 3 | | | | Comparability: 0 | Outcome: 3 | | | | |  |
| Eid et al. | 1 | 1 | 1 | 1 | 0 | 1 | 0 | | 0 | | **5** |
|  | Selection: 4 | | | | Comparability: 0 | Outcome: 1 | | | | |  |
| Williams et al. | 0 | 1 | 1 | 1 | 0 | 1 | 1 | | 1 | | **6** |
|  | Selection: 3 | | | | Comparability: 0 | Outcome: 3 | | | | |  |
| McCreary et al. | 1 | 1 | 1 | 1 | 2 | 1 | 0 | | 0 | | **7** |
|  | Selection: 4 | | | | Comparability: 2 | Outcome: 1 | | | | |  |
| Tumash et et al | 0 | 1 | 1 | 0 | 0 | 1 | 1 | | 1 | | **5** |
|  | Selection: 2 | | | | Comparability: 0 | Outcome: 3 | | | | |  |
| Sinchikhin et al. | 0 | 1 | 1 | 1 | 0 | 1 | 1 | | 1 | | **6** |
|  | Selection: 3 | | | | Comparability: 0 | Outcome: 3 | | | | |  |
| Valsecchi et al. | 1 | 0 | 1 | 1 | 0 | 1 | | 1 | | 1 | **6** |
|  | Selection: 3 | | | | Comparability: 0 | Outcome: 3 | | | | |  |
